# The *in vitro* activity of the tyrosine kinase inhibitor STI571 in BCR–ABL positive chronic myeloid leukaemia cells: synergistic interactions with anti-leukaemic agents

**DOI:** 10.1038/sj.bjc.6600288

**Published:** 2002-05-06

**Authors:** W M Liu, L A Stimson, S P Joel

**Affiliations:** Barry Reed Oncology Laboratory, 4th Floor, 38 Little Britain, St. Bartholomew's Hospital, West Smithfield, London EC1A 7BE, UK

**Keywords:** STI571, BCR–ABL, tyrosine kinase, etoposide, cytarabine

## Abstract

Chronic myeloid leukaemia is typically characterised by the presence of dysregulated BCR–ABL tyrosine kinase activity, which is central to the oncogenic feature of being resistant to a wide range of cytotoxic agents. We have investigated whether the inhibition of this tyrosine kinase by the novel compound STI571 (formerly CGP57148B) would render K562, KU812 cell lines and chronic myeloid leukaemia-progenitor cells sensitive to induction of cell kill. Proliferation assays showed STI571 to be an effective cytotoxic agent in chronic myeloid leukaemia-derived cell lines (IC_50_ on day 5 of 4.6 μg ml^−1^ and 3.4 μg ml^−1^ for K562 and KU812 respectively) and in leukaemic blast cells (per cent viability on day 3 at 4 μg ml^−1^: 55.5±8.7 *vs* 96.4±3.7%). STI571 also appeared to specifically target *bcr–abl* expressing cells, as results from colony forming assays using the surviving cell fraction from STI571-treated peripheral CD34^+^ chronic myeloid leukaemia blast cells, indicated a reduction in the expansion of colonies of myeloid lineage, but no effect on normal colony formation. Our data also showed synergy between STI571 and other anti-leukaemic agents; as an example, there were significant increases in per cent cell kill in cell lines cultured with both STI571 and etoposide compared to the two alone (per cent cell kill on day 3: 73.7±11.3 *vs* 44.5±8.7 and 17.8±7.0% in cultures with STI571 and etoposide alone respectively; *P*<0.001). This study confirms the central oncogenic role of BCR–ABL in the pathogenesis of chronic myeloid leukaemia, and highlights the role of targeting this tyrosine kinase as a useful tool in the clinical management of the disease.

*British Journal of Cancer* (2002) **86**, 1472–1478. DOI: 10.1038/sj/bjc/6600288 www.bjcancer.com

© 2002 Cancer Research UK

## 

We have previously demonstrated the human chronic myeloid leukaemic (CML) cell lines K562 and KU812 to be relatively resistant to apoptosis induced by a wide range of anti-cancer agents, including etoposide and cisplatin (Liu *et al*, 2002). Although the exact mechanism of this aberrant suppression of apoptosis is poorly understood, studies have highlighted the importance of the Philadelphia chromosome present in these cell types (reviewed in Holyoake, 2001). This balanced reciprocal translocation fuses together the c-*abl* proto-oncogene from chromosome 9 to the breakpoint-cluster region of the *bcr* gene on chromosome 22 (Nowell and Hungerford, 1960; Rowley, 1973).

Mammalian C-ABL belong to a family of tyrosine kinases (TK), the biological function of which remains unclear, although it has been shown to have a diverse role in the regulation of multiple cellular processes including transcription, DNA repair and the cell cycle. The *bcr–abl* gene created by this inter-chromosomal exchange encodes one of two fusion proteins, p185 and p210, that display elevated and dysregulated TK activity, and forms the fundamental mechanism underlying CML positive cells. The p210 form of BCR–ABL is seen in 95% of all patients with CML and up to 20% of adult patients with acute lymphocytic leukaemia (ALL) (Bartram *et al*, 1983; Shtivelman *et al*, 1985), whereas the p185 form is seen in 5–10% of *de novo* ALL (Hermans *et al*, 1987; Clark *et al*, 1988). Currently, allogeneic bone marrow transplantation is the only proven curative therapy for CML, and the cure rate for approximately one-third of CML patients that are eligible is 70–90% (Kantarjian *et al*, 1993; Goldman, 1994). Interferon-α has been reported to induce both sustained remissions (Sanchez *et al*, 1992) and prolong survival (Allan *et al*, 1994; NCI, 1997), however, this is not a curative therapy. As it is generally accepted that TK activity is required for the transforming ability of BCR–ABL fusion-proteins (Lugo *et al*, 1990; Oda *et al*, 1995), a specific inhibitor of TK function may represent a possible therapy for CML and other *bcr–abl* positive leukaemias (Levitzki and Gazit, 1995).

One such class of TK inhibitors, referred to as tyrphostins, was reported in the late 1980s (Yaish *et al*, 1988). Synthesised and developed by a team in Israel, this novel class of compounds was one of the first to demonstrate p210 BCR–ABL TK inhibitory properties, inducing cell kill in K562 cells (Anafi *et al*, 1993). Recently, a novel 2-phenylaminopyrimidine derivative named STI571 (formerly CGP57148B) has been reported, which has improved specificity over the tyrphostins (Buchdunger *et al*, 1996; Druker *et al*, 1996). The compound was designed in 1996 by using the known structure of the ATP binding site of protein kinases, and early biochemical screening showed STI571 to be a potent inhibitor of BCR–ABL TK (IC_50_: 0.025 μM *in vitro* for substrate phosphorylation). The results of cell line experiments showed STI571 was capable of selectively and effectively inhibiting the growth of *bcr–abl* positive cell lines (K562), whilst appearing to have no affect on the proliferation of cell lines expressing other TKs such as v-*src* (Druker *et al*, 1996). Similarly, colony-forming assays also showed STI571 specifically inhibited the formation of *bcr–abl* positive colonies by 90%, without affecting *bcr–abl* negative cells. *In vivo* data from murine experiments showed limited activity at >10 mg kg^−1^; however, complete cures were not reported. A number of phase I trials by Druker investigating the efficacy of STI571 in CML patients presenting at different stages of the disease have shown good results. The initial study included 83 patients with chronic phase disease who had failed interferon-α therapy. The minimum effective dose was 300 mg, producing complete haematological response in 98% (Druker *et al*, 2001a). In another study of 233 patients with accelerated phase CML, there were responses in 91% of patients treated, of which 63% were complete responses (Mauro and Druker, 2001). The effectiveness of STI571 in the earlier stages of the disease resulted in an expansion of these phase I studies to include CML patients in myeloid and lymphoid blast crisis and patients with relapsed or non-responsive BCR–ABL positive ALL. Responses occurred in 21 of 38 patients (55%) with myeloid blast crisis, and in 14 of 21 patients (70%) with lymphoid crisis or ALL (Druker *et al*, 2001b). Phase II and III studies are currently ongoing in newly diagnosed patients.

This report details the *in vitro* effects of single-agent STI571 in CD34^+^ CML stem cells and CML-derived cell lines. Interactions with the cytotoxic agents etoposide and cytarabine were also assessed.

## MATERIALS AND METHODS

### TK inhibitor – STI571

The 2-phenylaminopyrimidine derivative designated STI571 was kindly provided by Novartis Inc (Basel, Switzerland). A 1 mg ml^−1^ (≈1.7 mM) stock solution in dimethylsuphoxide (DMSO; Sigma Ltd, Dorset, UK) was prepared from the total of 10 mg provided, of which appropriate working dilutions were made prior to each experiment.

### *In vitro* analysis – cell lines

K562 and KU812 cell lines were maintained in RPMI-1640 medium supplemented with 10% FBS and 1% PS, in a humidified atmosphere with 5% CO_2_ in air at 37°C.

To study the effect of a continuous exposure, K562 and KU812 cells (2×10^5^ cell ml^−1^) growing exponentially were cultured for 5 days with STI571 at a range of concentrations between 0–5 μg ml^−1^. Aliquots were removed daily for assessment of viability by Trypan blue exclusion and cell cycle distribution including apoptosis.

To study the effect of STI571 in combination with existing cytotoxic agents, K562 and KU812 cells (2×10^5^ cell ml^−1^) were cultured for 5 days with either 0.8 μM etoposide or 40 nM cytarabine (both Sigma) in the presence or absence of 4 μg ml^−1^ STI571 (≈IC_50_). Cell counting and cell cycle analysis were performed daily. As these cell lines were resistant to a continuous exposure to these cytotoxic agents, IC_50_ values for viability could not be determined. Therefore, the concentrations were chosen based on their ability to inhibit cell proliferation by about 50%.

All cell counts were expressed as population doublings (PD) using the following formula:


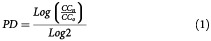


where *n*=day of study.

### *Ex vivo* analysis – CD34^+^/CML-positive blast cells

CD34^+^ stem cells from the peripheral blood of four patients with CML in blast crisis were harvested by using anti-CD34^+^ conjugated magnetic beads. All patients gave informed consent to the collection of an additional 10 ml blood sample at the same time as venesection for routine pathology, and all samples were coded to conceal the patients' identity. The purity of enrichment ranged between 83–91%. Cells (2×10^5^ cell ml^−1^) were washed and reset in RPMI−1640 medium supplemented with 10% FBS and 1% PS and maintained at 37°C in a humidified atmosphere with 5% CO_2_. Cell mitogenic activity was stimulated with phytohaemagglutinin-M (PHA-M; Sigma) at 20 μg ml^−1^. Following an initial 6-h incubation, one of three drug combinations were added, STI571 alone (4 μg ml^−1^); etoposide alone (0.5 μM); combination (4 μg ml^−1^ STI571 and 0.5 μM etoposide). Aliquots were removed daily for analysis of cell proliferation and viability by Trypan blue analysis. In addition, aliquots were removed on day 3 for clonogenic assays.

### Haemopoietic clonogenic assays

Low-density mononuclear cells (MNC) from haematologically normal donors, harvested through fractionation with Histopaque-1077 (Sigma) and cells harvested from the preliminary *ex vivo* suspension cultures were re-suspended in RPMI-1640 medium. Cells (1×10^5^ cell ml^−1^) were plated in methylcellulose cultures (Stem Cell Tech., USA) containing 0.9% methylcellulose and 30% FBS. Cultures were supplemented with a combination of SCF (100 ng ml^−1^ – Amgen Ltd, Cambridge, UK), IL-3 (100 ng ml^−1^ – Sandoz Pharmaceuticals, Frimley, UK), GM-CSF (50 ng ml^−1^ – Sandoz) and EPO (2 U ml^−1^ Janssen-Cilag Ltd, High Wycombe, UK). Culture dishes were incubated at 37°C in a humidified atmosphere with 5% CO_2_. Colony formation was assessed and typed after 14-days according to published methods (Eaves and Eaves, 1984).

### DNA analysis

The distinct phases of the cell cycle were distinguished by flow cytometry. Cells were washed in ice-cold nucleus buffer (0.15 M NaCl, 5 mM MgCl_2_, 1 mM KH_2_PO_4_, 1 mM EGTA, 0.1 mM Dithiothreitol, 10% glycerol in distilled water pH 6.5). Cells (1×10^6^) were re-suspended in 4 ml of freshly constituted permeabilising solution (0.35% Triton X-100, 0.1 mM PMSF in nucleus buffer), and mixed by rotation at 4°C for 20 min. Cells were then fixed by adding 4 ml of ice-cold methanol and rotated for a further 30 min. Samples were washed with ice-cold PBS before staining with 500 μl of PI stain (50 μg ml^−1^ propidium iodide and 50 μg ml^−1^ RNAse A in PBS). Acquisition of data was performed within 1 h using a Becton Dickinson FACScan machine. Ten thousand cells were analysed for each data point, and the percentages of cells in sub-G1 (apoptotic fraction), G1, S and G2/M phases were analysed using a cell cycle analysis program (WinMDI 2.4).

### Assessment of p21^waf1^ by immunoblot analysis

Whole cell lysates were resolved by SDS–PAGE using 15% acrylamide with a 5% stacking gel as described previously (Liu *et al*, 2002). Briefly, primary antibody probing was performed with mouse-anti-p21^waf1^ at a concentration of 0.2 μg ml^−1^ (PharMingen). Mouse anti-β-actin was used as a loading control (1 : 2000, Oncogene Research Products, Boston, USA). Following a washing step in 0.1% Tween in tris buffered saline (Sigma, 100 mM Tris, 150 mM NaCl, pH 7.6), horseradish peroxidase-conjugated anti-mouse IgG was used as the secondary antibody (DAKO Ltd, High Wycombe, UK). Bands were visualised by the ECL plus detection system (Amersham Life Science Ltd, Little Chalfont, UK).

### Statistical analysis

All statistical analyses were carried out using Minitab version 10 (State College, PA, USA). Control samples were normally distributed as determined by the Shapiro-Wilk test, and parametric tests were used throughout. Any difference between variables and control cultures, as determined by ANOVA, were further characterised by the standard paired Student's *t*-test.

The concentration of any of the tested agents required to cause a 50% reduction in cell viability or cell number was determined with an adapted version of the sigmoid E_max_ model as shown:


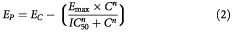


where *E_P_*=predicted effect; *E_C_*=control effect; *E_max_*=maximum effect; *C*=concentration of drug; *n*=sigmoid-fit factor (Holford and Sheiner, 1981).

## RESULTS

The percentage viability of cells in control cultures was >88% at all time points. Separate experiments in stem cells harvested from the bone marrow of haematologically normal volunteers revealed that optimal colony formation was achieved using a SCF, IL-3, GM–CSF and EPO cocktail, consequently, all short-term cultures utilised this combination (McGuckin *et al*, 1996).

### STI571 reduces the viability of CML-derived cell lines

**Figure 1:**
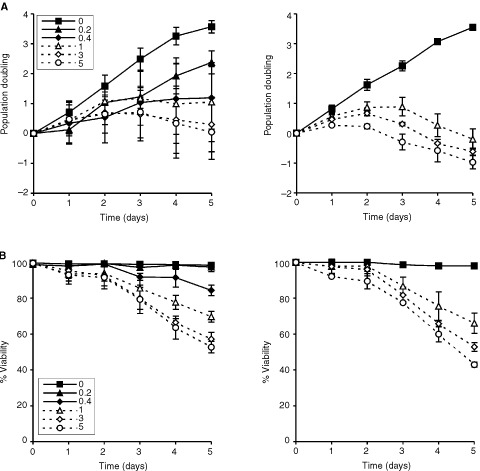
(**A**) Effect of STI571 on PD in K562 (left) and KU812 (right) cells. Points represent the mean and s.d. of three separate experiments. (**B**) Effect of STI571 on per cent viability K562 (left) and KU812 (right) cells. Points represent the means and s.d. of three separate experiments.

A concentration and duration-dependent reduction in the rate of cell proliferation expressed as a population doubling (PD), was observed in both cell lines cultured with STI571 continuously for 5 days (Figure 1A

). A decrease in viability, which reached significance at the higher concentrations and durations in both cell lines, mirrored this reduction in PD (Figure 1B). The concentration of the STI571 resulting in 50% reduction in per cent viability on day 5 (IC_50_) was calculated to be 4.9 μg ml^−1^ (≈8.4 μM) and 3.5 μg ml^−1^ (≈5.9 μM) for K562 and KU812 respectively.

**Figure 2:**
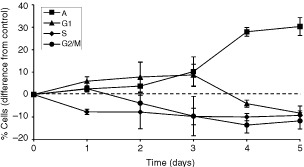
Effect of 5 μg ml^-1^ STI571 on cell cycle distribution over 5-days in K562 cells. Points represent the means and s.d. of four separate experiments.

Flow cytometric analysis showed an increased number of cells undergoing apoptosis in cultures with STI571, with concomitant reductions in the other phases of the cell cycle. This was most clearly demonstrated in K562 cells treated with 5 μg ml^−1^ STI571 (Figure 2

)

### STI571 increases the cytotoxic effect of etoposide in K562 and KU812 cells

**Figure 3:**
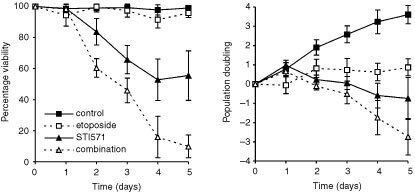
Effect of STI571 and 0.8 μM etoposide on per cent viability and PD in K562 cells. Points represent the means and s.d. of three separate experiments.

**Figure 4:**
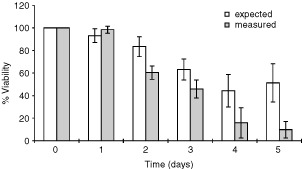
Viability in K562 cells treated with a combination of STI571 and etoposide (measured) were compared to the expected per cent viability. This was calculated as the sum of the per cent viability in cells treated with either STI571 or etoposide (see text). Points represent the means of six separate experiments.

In K562 cells, single-agent etoposide (0.8 μM) had no cytotoxic effect at any time throughout the 5 day culture. Instead, a cytostatic response was seen, as indicated by a reduction in the rate of PD by day 2 (day 2: 0.8±0.5 *vs* 1.9±0.4 in control cells; *P*<0.001) (Figure 3

), and an increasing block in G2 phase of the cell cycle. As described in the previous section, the continuous exposure of K562 cells to STI571 resulted in a significant reduction in per cent viability by day 3. Most interestingly, the combination of etoposide with STI571 resulted in a synergistic cytotoxic effect, with greater reduction in cell viability than expected. This was clearly demonstrated by comparing the total per cent viability in cultures with a combination of STI571 and etoposide with the numerical sum of per cent viability in cultures with the two drugs separately (Figure 4

). As an example, on day 5, per cent viability in K562 cells cultured with etoposide alone was 95.8%, and 55.5% with STI571 alone. Numerically, the decrease in per cent viability in cells cultured with a combination of these two drugs would be 48.7%, resulting in an expected per cent viability of 51.3%, the sum of the viabilities. However, the observed per cent viability of cultures with both drugs was 9.9%, indicating a hyper-additive (synergistic) effect (*P*<0.05).

Results in KU812 cells showed essentially the same pattern of response, with significantly decreased cell viability in cells treated with a combination of both etoposide and STI571 compared to the drugs individually (per cent viability on day 5: 34.1±1.4 *vs* 95.9±3.7 and 50.0±3.8% respectively; *P*<0.001).

**Figure 5:**
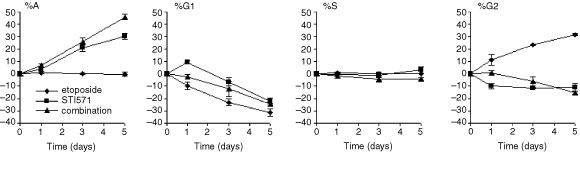
Effect of STI571 and etoposide on the cell cycle of KU812 cells. Cells were treated with either etoposide alone, STI571 alone or a combination of the two. Points represent the means and s.d. of three separate experiments.

Subsequent flow cytometric analysis in these cells indicated cell kill to be partially by apoptosis as there was a concomitant increase in %A. Parenthetically, %A on day 5 in K562: 5.0±1.8, 32.9±5.7 and 38.0±5.9% in etoposide alone, STI571 alone and in combination, respectively (KU812 results shown in Figure 5

). Interestingly, the block in the G2 phase of the cell cycle as seen in cultures with etoposide alone was clearly absent in cultures containing both etoposide and STI571 (Figure 5).

### p21^waf1^ levels are not increased in STI571-treated K562 cells

**Figure 6:**
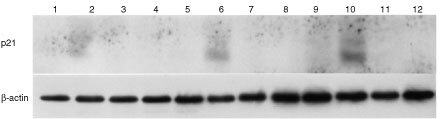
p21 levels in K562 cells treated with etoposide alone (lanes: 2, 6, 10), STI571 alone (lanes: 3, 7, 11) or a combination of the two (lanes: 4, 8, 12). Cells were treated continuously for 5 days. Samples from day 3 (lanes: 1–4), day 4 (lanes: 5–8) and day 5 (lanes: 9–12) are shown, with respective controls (lanes: 1, 5, 9).

Whole cell lysates from K562 cells treated with STI571 alone and in combination with etoposide were separated by electrophoresis, and then immunoprobed for p21^waf1^ protein. Results indicated increased levels in cells cultured with etoposide alone but not in the other samples. This pattern of p21^waf1^ expression and drug-combination was seen consistently on days 3–5 (Figure 6

).

### STI571 increases the cytotoxic effect of cytarabine in K562 and KU812 cells

The results seen in K562 and KU812 cells were essentially the same, so only results from K562 experiments are presented. Cytarabine alone had no significant effect on cell viability over the 5-day period (per cent viability day 5: 94.6±3.6 *vs* 97.6±0.8% in control cells), however, the combination of cytarabine with STI571 produced a significant increase in the loss of viability compared to just STI571 alone (*P*<0.001) (Table 1

**Table 1:**
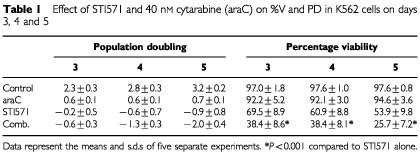
Effect of STI571 and 40 nM cytarabine (araC) on %V and PD in K562 cells on days 3, 4 and 5

).

### STI571 reduces the viability of CD34^+^ CML blast cells

Figure 7

**Figure 7:**
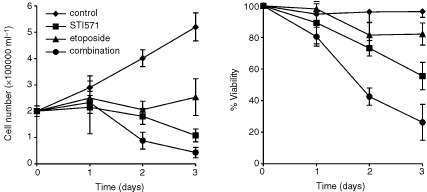
Effect of STI571 and etoposide on per cent viability and PD in CML blast cells. Points represent the means and s.d. of three separate patients.

highlights the effect of etoposide and STI571 on cell proliferation and viability in CD34^+^ CML blast cells.

In accordance with previous results from our *in vitro* experiments, etoposide had a cytostatic effect as indicated by an inhibition of cell proliferation, with only small decreases in PD. Conversely, in cultures with STI571 alone, there was a reduction in cell number and a concomitant duration dependent decrease in per cent viability. Most notably, however, there was a greater loss of viability in cells treated with both drugs compared with etoposide or STI571 alone, which correlated with the cell line data.

A synergistic effect was seen between STI571 and etoposide, as comparison of the difference in per cent cell kill in cultures using the drugs simultaneously with the expected value, revealed consistently more per cent cell kill in the former (difference in cell kill on days 1, 2 and 3: ^+^6.9±5.5, ^+^12.2±7.8 and ^+^11.4±6.9% respectively), which was significant on days 2 and 3 (*P*=0.05)

### STI571 increases the colony forming potential of CML blast cells

CD34^+^ CML cells that were cultured with drugs for 3 days were plated in short-term semi-solid cultures supplemented with a cocktail of GFs. Colony formation was typed and enumerated on day 14. This allowed for an assessment of the effect of STI571 on surviving progenitors following culture in suspension.

The total number of colony and burst forming units (CFUs and BFUs) developing from the blast cells of haematologically normal volunteers cultured with GFs had been previously assessed as 171.5±6.7, and the distribution of colony type was evenly divided between myeloid and erythroid (45.5% CFU-G, 10.5% CFU-M, 3.2% CFU-GM, 40.7% BFU-e). In control plates of CML cells with PHA-M alone, there were large numbers of individual cells (non-colony forming), and only 47.0±6.4 observable colonies that were exclusively of the CFU-G type. A similar pattern of cell and colony proliferation was seen in PHA-M-stimulated blast cells that had been treated with etoposide alone of just 41.7±3.5 colonies (97.9% CFU-G).

Most interestingly, those cultures of cells that had been treated with STI571 alone showed increased numbers of colony forming cells, with a total of 62.7±2.1 colonies that was also made up of non-CFU-G types (81.9% CFU-G, 13.9% CFU-M, 4.1% CFU-GM). Cells treated with both drugs produced a total colony count that was significantly higher than controls, but similar to those with STI571 alone (66.7±8.5 *vs* 47.0±6.4 and 62.7±2.1 respectively; *P*<0.001 in the former comparison). Additionally, there was a lower ratio of CFU-G to non-CFU-G colonies produced from these cells compared to cells treated with STI571 alone (66.1% CFU-G, 21.6% CFU-M, and 12.3% CFU-GM) (*P*=0.07).

## DISCUSSION

We have previously shown the CML-derived cell lines K562 and KU812 to be resistant to a range of drugs. Although the exact mechanism of this resistance has yet to be fully elucidated, there is strong evidence suggesting that the constitutive expression of BCR–ABL protein TK in these cell lines contributes to the phenotype. This study was therefore undertaken to investigate the importance of functional BCR–ABL TK in determining the sensitivity of CML-derived cell lines to anti-cancer agents. To this end, CML-cell lines and CD34^+^ blast cells harvested from CML patients, were cultured with a TK inhibitor (STI571) both alone and in combination with other anti-leukaemic agents. Not only did we show in cell lines and in blast cells that STI571 was an effective cytotoxic as a single agent, but we also showed synergistic interaction with etoposide and cytarabine.

In the first part of our investigation, we determined the effect of a continuous exposure to STI571 on cell proliferation and viability in K562 and KU812 cell lines. Calculation of IC_50_ suggested KU812 to be possibly more sensitive to STI571 than K562 (5.9 μM *vs* 8.4 μM). Results indicated clear concentration and duration dependent decreases in cell viability in both cell lines. Additionally, subsequent flow cytometric analysis showed that cell kill was by apoptosis, occurring from all phases of the cell cycle. These IC-values were higher than those reported in recent synergy studies (Thiesing *et al*, 2000; Kano *et al*, 2001; Topaly *et al*, 2001). Methodologically, the activity of STI571 in combination in these studies was assessed by the MTT assay, which is strictly a technique for measuring cell number and not cell kill (Carmichael *et al*, 1987). Consequently, as the assay cannot distinguish between cytostasis and cytotoxicity, these two distinct processes are grouped as the same, resulting in a greater level of activity than would otherwise have been seen if viability was measured exclusively (Hoffman, 1991).

We next investigated the effect of combining STI571 with the anti-leukaemic agents etoposide and cytarabine in both K562 and KU812. The effect of culturing cells with either agent alone was solely one of cytostasis. However, the culture of cells with either drug in combination with STI571 resulted in increased apoptosis and significantly reduced cell viability. In particular, the expected block in G2/M induced by both etoposide and cytarabine was not seen in cells when cultured with STI571 and either drug. As the level of p21^waf1^ expression is important in mediating this G2/M block and in predicting cytotoxicity (Liu *et al*, 2002), the levels of this protein were assessed in K562 cells. Interestingly, the levels in cells cultured with STI571, either alone or in combination with other cytotoxics, was unchanged from that seen in controls.

Following the results of our cell line experiments, the effect of STI571 on cell growth was modelled and studied further in CML-derived blast cells. CD34^+^ mononuclear progenitor stem cells (PSCs) were harvested from the peripheral blood of CML patients, mitogenically stimulated, and then cultured for 3-days in the presence of STI571, etoposide or a combination of the two. As with the cell line studies, there was a duration dependent decrease in cell viability in PSCs cultures treated with STI571 alone. Also, in accordance with cell line data, etoposide had a cytostatic effect on PSCs, with viability remaining relatively high. Most importantly, the combination of STI571 with etoposide also showed a greater reduction in cell viability compared to either of the drugs alone.

Mechanistically, STI571 has been shown to inhibit Akt kinase through its interactions with BCR–ABL (Fang *et al*, 2000). Akt kinase is a key component of the apoptotic cascade, and can interact at many stages. It has been shown to phosphorylate and inactivate BAD (Datta *et al*, 1999), by converting it to an inactive moiety that is bound to the cytosol, and is therefore unable to hetero-dimerise with BCL-2 and BCL-X_L_ (Yang *et al*, 1995). Cell survival signals are thus promoted. Akt kinase has also been shown to directly phosphorylate and inactivate caspase-9, thereby preventing the activation of the executioner caspases (Cardone *et al*, 1998). Consequently, the inhibition of Akt kinase can result in a sensitisation of cells to apoptosis by existing chemotherapeutic agents by down-regulating BCL-X_L_ and enhancing the caspase cascade.

To further characterise the effects of STI571 on PSCs, the clonogenicity of the surviving fraction of committed progenitor cells were analysed in colony-forming assays. A hallmark of CML is the expansion of immature progenitor cells of the myeloid lineage in the bone marrow compartment, so that the majority of peripheral circulating CD34^+^ cells from CML patients are myeloid (Fialkow *et al*, 1977). PSCs were harvested from suspension cultures, washed and plated on semi-solid methylcellulose plates supplemented with a cocktail of GFs conducive to erythroid colony formation (McGuckin *et al*, 1996). Results were similar to those of Thiesing *et al* (2000), and showed that although there was a large number of cells seen on the culture plates, most lacked clonogenic capability since only a small number of colonies were seen. As expected of blast cells from CML patients with abnormal haemopoiesis, there was a reduction in the total number of colonies, which were all of the CFU-G class. The intrinsic nature of etoposide cytotoxicity, which is dependent upon the levels of the nuclear enzyme topo II (Zwelling, 1985), would have an equal effect in both the normal and abnormal surviving PSCs. This was reflected in our clonogenic assays that were essentially the same as those seen in control cultures with no added drug, having only CFU-G colonies present. Interestingly, the culture of PSCs that had been treated with STI571 resulted in an overall increase in the total number and heterogeneity of colonies, indicating the presence of a surviving fraction of normal haemopoietic progenitor cells and thus some drug specificity. The combination of STI571 and etoposide produced even greater numbers of non-CFU-G colonies, indicating an advantage of co-culture with existing cytotoxics.

Overall, our findings are in general agreement with similar reports of drug interactions between STI571 and anti-leukaemic agents that have been published in the last year (Thiesing *et al*, 2000; Kano *et al*, 2001; Topaly *et al*, 2001). While these studies only stopped at highlighting a synergistic reduction in absolute cell number, we went on to specifically identify hyper-additive increases in apoptosis. In the present study, we only used simultaneous exposure to both STI571 and other agents. Numerous reports have highlighted the importance of drug scheduling in determining the ability to induce a cytotoxic response. Specifically, that the efficacy of agents that act on specific phases of the cell cycle can be improved by a sequential administration with STI571 (Mani *et al*, 2000; Senderowicz, 2001). Having demonstrated a synergistic benefit of the co-administration of STI571 with other anti-leukaemic agents, further studies are required to elucidate the precise mechanism of this enhancement, so that its application may be fully maximised.
